# Mental Therapy

**Published:** 1896-04

**Authors:** Matthew M. Smith

**Affiliations:** Austin, Texas


### TEXAS MEDICAL JOURNAL.

ESTABLISHED JULY, 1885.

PUBLISHED MONTHLY.—SUBSCRIPTION $2.00 A YEAR.

VOL. XI. AUSTIN, APRIL, 1896. NO. 10.

### Original Contributions.

For the Texas Medical Journal.

### MENTAL THERAPY.

BY MATTHEW M. SMITH, M. D., AUSTIN, TEXAS.

Read at meeting of Austin District Medical Society, March 19, 1896, and at the Texas Academy of Sciences, March 1.

MR. PRESIDENT, and Members of the Society:

"This is an unweeded garden, that grows to seed;
Things rank and gross in nature, possess it merely."

At one time the people proclaim the great panacea is faith-healing and Christian science. Again, the newspaper sand brass bands herald hypnotism and magnetic-healing as the sole benefactor; or quietly we are urged to put our faith in spiritualism, or some other belief, if we would be free from all ills.

But to whatever extent the legitimate practice of medicine fails to come up to the needs of the public, or to gratify the curiosity of the people at large; to that extent does charlatanism thrive, and quackery finds a congenial soil. Something new, mysterious and wonderful is what the people of to-day demand. But it must be admitted that no error can long be used with success, unless it has some element of truth as a basis; and the success attendant upon such absurdities as "Cure by the royal touch, the mad-stone, and the laying on of hands, and the annointing with oil," must certainly show the wonderful influence of the mind over the body.

My object for presenting this paper before the Academy, is to emphasize the great value of the mental factor in the production and cure of diseases; to show that educated and intelligent physicians do not hesitate to make use of it, as a means of treatment, whenever it promises to be of value; and lastly, to remind you that the day has certainly arrived when the individual should learn to be careful in the selection of his medical advisor; since legislation is sadly at fault in this and many other States.

I shall not attempt to say the whole mystery of life has been revealed, but during the last few years the microscope has done much toward making it clear, and it is well known; the phenomena of animal existence, is the combined result of individual lives of the microscopic cells of which all the various elements of the living body are composed. These cells exist in groups, forming organs which have definite functions to perform; one set composes the brain and produces thought; another the muscles-producing movement; a third, the transmitter of orders and commands, and when all are acting harmoniously, and each performing its individual function properly, the whole body is in a state of perfect health, and the highest principle that should guide the efforts of the medical profession, *is the production and preservation of a sound mind and a sound body.*

Dr. Maudsley says: "So intimate and essential is the sympathy between all the organic functions, of which the mind is the crown and consummation, that we may justly say of it, that it sums up and comprehends the bodily life;—that everything which is displayed outwardly is contained secretly in the innermost. We can not truly understand the mind functions without embracing in our inquiry all the bodily functions, and I might, perhaps, without exaggeration, say all the bodily features." He further says: "To me it seems not unreasonable to suppose that the mind may stamp its tone, if not its very features, on the individual elements of the body, inspiring them with hope and energy or infecting them with despair and feebleness." * "There is not a natural action in the body, whether voluntary or involuntary, that may not be influenced by the peculiar state of the mind at the time." † "It is impossible to be seized by a vivid idea, without the whole body being placed in harmony with this idea."

We have the tear of grief, the eye of pride, the frown of

* Dr. John Hunter.

† Gratiolet.

anger, the blush of embarrassment and the stare of astonishment. The typical expressions of the criminal, the idiot, the martyr, the saint. or the man of learning, all show, but too clearly, the mind's impression upon the bodily frame work.

The stick of a pin causes a message to be transmitted along a sensory nerve to the brain, where the idea of pain is formed, or *sensation acts on ideation*, and the reverse may be true, oftentimes, that *ideation can produce sensation.* This goes far towards explaining the great mental cause of disease. There are no imaginary diseases, but there are diseases due to the imagination, which may be accompanied by real functional disturbances. They are developed by the influence of suggestion in some form, and may be benefited or cured by the same element if rightly used.

Suggestion is the great element in mind treatment. It is the influence exerted by an idea suggested and received by the mind. Suggestion has revolutionized psychology; it is now being recognized in medico-legal questions. In the education of the child this element has a powerful influence. Sectarian schools and definite religious and political beliefs, realize its value and utilize it in every available way.

* Without being aware of it, we acquire moral and political predispositions, prejudices, etc. We are impregnated with the mental atmosphere about us; we honestly believe and defend as we would our own welfare, social and religious principles which may be opposed to common sense, not to say reason; these principles were held by our ancestors, they are also national, and descend from father to son. It is impossible to destroy them by argument, and dangerous to do so by force."

But all these elements of obscurity and doubt, are being gradually dispelled by the spread of scientific knowledge and truth, which demands a proof and a reason for every assertion and theory advanced. Since suggestion is purely a psychical influence, and as the roads to the centers of ideation and imagination are numerous, there are many forms of suggestion: by spoken words, reading, touch, surroundings, and auto-suggestion, viz.: impressions made by our own thoughts, which is a common cause for many diseased conditions. Suggestion exerts its influence in the wakeful state, even in the dreaming state, but best of all in the hypnotic state. It rules the greater part of the hypnotic manifestations, and the phenomena which are called

* Dr. Liebault.

physical, or only psychical, the hypnotized subject grapples the operator's thought; his brain is excited, and it is carried out through the means of an exalted suggestion, produced by the special concentration of mind in the hypnotic condition. Suggestion, then, introduces, cultivates, and confirms an idea in the mind of the subject; this idea forms the image, which produces a sensation, and the subject accepts, believes and acts upon it, thinking it original with himself.

* "Hypnotism is no longer one of the curiosities of science; it is a therapeutic resource of unquestionable value, and which lies at the hands of every medical practitioner who is willing to take the trouble to inquire into its nature and the manner of its use." The hypnotic state only places the patient in the most suitable condition for the powerful influence of suggestion, because his mind is not only in a state of hyper-receptivity, but, owing to the intensity of the suggestion, all of her thoughts or ideas make no conscious impressions, and therefore pass unnoticed. But such a condition should in no wise be considered mysterious or super-human, because we have manifestations of the same element daily in the wakeful state. The interested student may not feel the prick of the pin; the aching tooth is relieved by directing the attention elsewhere; marching is less wearisome if music engages the attention: the sight of game removes all the tired feeling of the hunter, and excitement of battle may prevent pain, even of serious gun-shot wounds.

We see the influence of these elements well demonstrated by traveling quacks. These fellows, with bands of music, and excitement, and well-lighted tents, extract teeth without pain. Magnetic healers and hypnotists, in crowded opera houses, with music and excitement, remove pains and stiffness from joints instantly, but temporarily; and the confirmed cripple throws down his crutch and walks, and even gives testimony in the morning paper; and the poor blind are made to believe they see better by the influence of this element. The wonderful cures reported from the "Shrine of the Black Virgin," the image of the infant Jesus, and a pilgrimage to the famous waters of "Lourdes" may be largely explained by the influence of implicit faith. continuous hope, and the use of mind treatment by suggestion. Stigmatization is a significant example of the powerful influence of the mind over the physical organism. Ceremonies, charms, gesticulations and amulets have in all ages and among

* Dr. Herter, of New York.

all nations been esteemed and greatly used in the treatment of disease, and the power is not super-human, but acts by strenghtening and exalting the imagination of the patient. Suggestion has a powerful influence over the bodily conditions; fear, anger, envy, anxiety, selfishness and sinful thought, all leave their effects upon the organism in the form of bodily expression; or "Mind translates itself into flesh." All are more or less familiar with the important role the imagination plays on disease; the patient is "not so sick as she is troubled with quick coming fancies that keep her from her rest."

Dr. Durand, of New Orleans, to test this mental influence, gave a hundred patients a dose of sweetened water; fifteen minutes after, entering the wards apparently in great excitement, he announced that he had by mistake given a powerful emetic, and preparations must be made accordingly; eighty out of the hundred patients soon fell to vomiting. Station men along the route of a susceptible individual; let each in turn refer to his sickly appearance, and the strong healthy man can be made to take his bed.

Special diseased organs will produce certain typical mental symptoms. See the hope, life and determination in lung cases on the verge of death; the despondency, fear and anxiety of heart trouble; the anger, envy and irritability when the liver is at fault; and so it is with cases involving any special organ of the body, the psychical phenomena will largely indicate the organ at fault, and upon this thoery "*sight* diagnosticians" claim to do wonders and appear as marvelous, but they only do what all intelligent physicians should do; except they see little, guess much, and make believe everything. A series of questions, a physical examination in detail, and many other adjuncts, certainly can do no harm toward diagnosing correctly, the extent and kind of lesion upon which an individual life depends, and which certainly is the only key to rational treatment.

If due credit is given the mental factor in the treatment of diseased conditions, you will have an explanation for many seemingly wonderful cures. Take, for instance, the "Keely Cure" for tobacco, opium and alcohol habits. What are the factors here to be considered? The victim realizes fully his deplorable condition; he knows his appetites have control of his will power; fear causes him to seek advise, because degradation and death stare him in the face; faith exerts its mysterious influence, or else he would not ask for treatment; his removal from associates, development of will power, destruction of habits, increase of faith and stimulation by medicine, all are sufficient to produce some cures and cause many temporary benefits.

The most successful physician is he, who, inspiring the greatest confidence in his remedies, strengthens and exalts the imagination of his patient. Bread pills, coated with hope and taken with confidence, will often do more good than scientific treatment. The physician must deal practically with the thoughts, feelings and conducts of his patients, and regard the mind, not as an abstract entity for speculation, but as a powerful force in nature, and the operations of which he must observe closely, patiently, and labor to influence beneficently, and acknowledge the essential unity of body and mind. Fear and faith of all the mysterious processes of the mind exert the greatest influence over the body. Fear predisposes to disease, and the exercise of simple faith will often cure it.

Confidence and faith in the physician has as much value as any drug in the pharmacopaea toward restoring normal function in many cases. We often hear the expression made to the physician: "Your visit does me more good than the medicine; when will you come again?" Daily mental gymnastics of ones well being for six months will prove a revelation to victims of insomnia, dyspepsia, nervous prostration, not to mention numerous other mental and physical conditions. But woe to the man who forms the habit of daily counting his pulse, and who watches closely his tongue, and if he gets a clinical thermometer and notes his temperature and selects some organ as his seat of trouble; such a man will soon seriously need the services of a physician, and he will be one of the most intractable cases for treatment, unless the physician is aided by the mind's influence. Much of the dread of surgical operations and the anxiety of child-birth, as well as numerous other trying ordeals can be avoided by keeping from the patients all sensational experiences of others, and by the use of well directed suggestions as to favorable results and speedy recoveries.

In presenting this paper before the Academy, I do not wish to be understood as not believing in the great therapeutic value of drugs, *for I do;* I am a firm believer in the rational method of medication, which has for centuries accumulated facts upon which the science and art now rest; which wages war against multiform disease by every possible means, and considers and uses any agent that can successfully prevent or combat disease in any of its forms, whether it comes from the Christian scientist, bacteriologist, homeopathist, chemist, or any other school or belief. True science will always accept well founded facts, and intelligent and educated physicians will make use of any element that will assist nature in her efforts to restore health, regardless of the source from which it originated; but those who act as lieutenants to mother nature in the restoration of health, should be well equipped in every detail. They should have a good education, well grounded on the sciences. They should study nature's laws and powers of resistance, consider the history of disease, including its ætiology, pathology, clinical history, treatment, etc., including the physiological action of drugs and the chemical and physiological laws which pertain to the peformance of the several functions; understand all the branches of medicine, and realize the reciprocal action between mind and body. All these will enable one to have a clear conception, good judgment, and be able to recognize, analyze, differentiate and classify symptoms, which only allow definite and rational conclusions and proper treatment. I have briefly enumerated many of the qualifications demanded by rational medicine, and it is certainly time the public should pay more regard to the difference between the honest, trustworthy and enlightened physician and the quack or "boastful pretender to an art he does not understand;" not for the benefit or the community at large, but for their own purse and life's sake.

I shall not take up the time by enumerating the classes of cases benefited or cured by mental therapy, but shall conclude with the following general advice, as applicable in using the mental elements in the treatment of disease: As far as you can consistently with truth, lead the patient to believe a given result will accrue, and you will have gone a long ways in reaching the end of your medication. Excite his will power, make him believe that much depends on his own determination, and always remember to administer the cordial of hope. Hope? Aye, who can measure its power to reanimate and vivify, to whip up the languid hearts, and send the blood coursing through the artery and vein; to drive the nerve fluid with electric speed, to awake into renewed activity every organ and tissue; to smooth, though with delusive touch, the pillow of disease and pain. Hope? Who, with magic wand, doth dispel our doubts and fears, and who doth line with silver sheen the dark cloud of despair.

"Hope? thou beggar's wealth,
Thou lover's victory, and thou sick man's health."

"What though the causes may not be explained,
Since these effects are daily ascertained,
Let not self-interest or pride,
Induce mankind to set the means aside;
Means, which though simple, are by heaven designed
To alleviate the woes of human kind."

